# Regulation of Cilium Length and Intraflagellar Transport by the RCK-Kinases ICK and MOK in Renal Epithelial Cells

**DOI:** 10.1371/journal.pone.0108470

**Published:** 2014-09-22

**Authors:** Joost R. Broekhuis, Kristen J. Verhey, Gert Jansen

**Affiliations:** 1 Department of Cell Biology, Erasmus MC, Rotterdam, the Netherlands; 2 Department of Cell and Developmental Biology, University of Michigan Medical School, Ann Arbor, Michigan, United States of America; Justus-Liebig-University Giessen, Germany

## Abstract

Primary cilia are important sensory organelles. They exist in a wide variety of lengths, which could reflect different cell-specific functions. How cilium length is regulated is unclear, but it probably involves intraflagellar transport (IFT), which transports protein complexes along the ciliary axoneme. Studies in various organisms have identified the small, conserved family of *ros*-cross hybridizing kinases (RCK) as regulators of cilium length. Here we show that Intestinal Cell Kinase (ICK) and MAPK/MAK/MRK overlapping kinase (MOK), two members of this family, localize to cilia of mouse renal epithelial (IMCD-3) cells and negatively regulate cilium length. To analyze the effects of ICK and MOK on the IFT machinery, we set up live imaging of five fluorescently tagged IFT proteins: KIF3B, a subunit of kinesin-II, the main anterograde IFT motor, complex A protein IFT43, complex B protein IFT20, BBSome protein BBS8 and homodimeric kinesin KIF17, whose function in mammalian cilia is unclear. Interestingly, all five proteins moved at ∼0.45 µm/s in anterograde and retrograde direction, suggesting they are all transported by the same machinery. Moreover, GFP tagged ICK and MOK moved at similar velocities as the IFT proteins, suggesting they are part of, or transported by the IFT machinery. Indeed, loss- or gain-of-function of ICK affected IFT speeds: knockdown increased anterograde velocities, whereas overexpression reduced retrograde speed. In contrast, MOK knockdown or overexpression did not affect IFT speeds. Finally, we found that the effects of ICK or MOK knockdown on cilium length and IFT are suppressed by rapamycin treatment, suggesting that these effects require the mTORC1 pathway. Our results confirm the importance of RCK kinases as regulators of cilium length and IFT. However, whereas some of our results suggest a direct correlation between cilium length and IFT speed, other results indicate that cilium length can be modulated independent of IFT speed.

## Introduction

Primary cilia are microtubule-based protrusions that can be found on the surface of almost all vertebrate cells, and function as sensory organelles. Defects in cilia function, structure or length have been associated with many genetic diseases, collectively called ciliopathies [1]. The assembly and maintenance of primary cilia depends on intraflagellar transport (IFT), a microtubule-based transport system that involves kinesin motor proteins (kinesin-II and KIF17/OSM-3) which mediate anterograde transport (to the tip of the cilium), dynein motor complexes (cytoplasmic dynein 2), which mediate retrograde transport (back to the cell body), and adaptor complexes (complex A, complex B, and the BBSome) [2].

A wide variety in cilia lengths and morphologies exist, most likely to better support cilium function in specific tissues [3], [4]. Although several signaling molecules have been shown to modulate cilium length [5]–[7], how this is achieved mechanistically is not understood. The most plausible explanation is provided by the balance point model, in which cilium length is determined by a balance between cilium assembly and disassembly rates [8], [9]. The assembly rate is dependent on the availability of axonemal tubulin and other structural components, supplemented by anterograde IFT and probably the pool of these proteins at the base of the cilium [10], [11]. Indeed, changes in both anterograde and retrograde IFT are accompanied by changes in cilium length [8], [12]–[15]. How cilium disassembly is regulated is unclear, since it seems independent of retrograde IFT [8], [15].

The family of *ros* cross-hybridizing kinases (RCKs) is characterized by a MAP kinase-like Thr-Xaa-Tyr (TXY) motif in their activation loop, and an overall structure similar to CDKs [16], [17]. In *Chlamydomonas* (*lf4*), *Leishmania* (*LmxMPK9*), *C. elegans* (*dyf-5*), and mouse (*Mak* and *Ick*) RCKs have been identified that negatively regulate cilium length [12], [18]–[23]. Emerging evidence suggests that regulation of cilium length may be manifest by RCK-induced changes in IFT. In *C. elegans, dyf-5* loss-of-function abolishes the coordination between the anterograde motors kinesin-II and OSM-3, such that the IFT complex travels with kinesin-II. In addition, kinesin-II can move into the distal segment in *dyf-5* mutant animals [12]. A more direct correlation between a change in IFT and cilium length was observed in recent studies in *Chlamydomonas* where *lf4* loss-of-function cells displayed an increased injection of IFT particles which correlates with increased flagellar assembly and length, and in mice where ICK was found to phosphorylate the kinesin-II subunit KIF3A and deletion of *Ick* affected the localization of IFT proteins in cilia [9], [14], [22].

In mammals, the RCK family contains three members: MAK or RCK (male germ cell-associated kinase, *ros* cross-hybridizing kinase), ICK or MRK (intestinal cell kinase, MAK-related kinase) and RAGE, MOK or STK30 (renal tumor antigen, MAPK/MAK/MRK overlapping kinase, serine threonine kinase 30) [17], [24]–[29]. MAK localizes to the connecting cilium and outer-segment axoneme in photoreceptor cells [20]. In retina of *Mak* knock-out mice cilia are elongated, IFT markers mislocalized, and photoreceptors degenerate over time [20]. In line with these observations, mutations in *MAK* have been found in patients with Retinitis Pigmentosa [30], [31]. Recently, it was shown that ICK localizes to primary cilia, inhibits ciliogenesis and regulates cilium length [21]–[23]. *Ick* knock-out mice show multiple developmental defects, correlating with ciliary and Shh signaling defects [22], [23]. ICK has been associated with endocrine-cerebro-osteodysplasia (ECO), a lethal recessive disorder with ciliopathy-like symptoms [32].

We set out to investigate the roles of RCK kinases in regulating cilium length in renal epithelial cells. We found that mouse inner medullary collecting duct cells (IMCD-3) express two of the three RCKs, ICK and MOK, which localize to cilia and negatively regulate cilium length. To analyze the effects of ICK and MOK on the IFT machinery, we set up live imaging of five fluorescently tagged IFT proteins: kinesin-II subunit KIF3B, complex A protein IFT43, complex B protein IFT20, BBSome protein BBS8 and kinesin KIF17. All five proteins moved at ∼0.45 µm/s in anterograde and retrograde direction, suggesting they are all transported by the same machinery. GFP tagged ICK and MOK also moved at approximately 0.45 µm/s, suggesting they are part of, or transported by the IFT machinery. Interestingly, whereas loss- or gain-of-function of ICK affected IFT speeds, MOK knockdown or overexpression did not. Finally, we found that the effects of ICK or MOK knockdown on cilium length and IFT depend on mTORC1 signaling.

## Materials and Methods

### Cell culture and transfections

IMCD-3 cells (CRL-2123, ATCC) were grown in DMEM/F10 medium supplemented with 10% FCS, penicillin (100 U/ml) and streptomycin (100 µg/ml). For transient transfections IMCD-3 cells, at 60% confluency, were transfected with FuGENE 6 (Roche), and serum starved for 48 hours to induce ciliogenesis. To generate clonal IMCD-3 cell lines, cells were transfected with linearized constructs. After 48 hours, G418 (500 µg/ml) was used to select transfected cells. After two weeks, viable GFP-positive cells were selected on a FACS Aria II cell sorter (Becton-Dickinson). Individual cells were seeded in a 96-well plate and cultured to confirm the GFP-construct expression levels and subcellular localization by fluorescence microscopy.

### Constructs

IFT43-YFP was a gift from Heleen Arts [33], and IFT20-GFP was a gift from Greg Pazour [34]. GFP-ICK was generated by PCR amplification of the ICK open reading frame (ORF) from mouse ICK cDNA clone (IMAGE 4224269) and subcloning into Clontech pEGFP-C1, using EcoRI and KpnI restriction sites engineered into the PCR primers. GFP-MOK was generated by amplification of the MOK ORF from mouse MOK cDNA clone (a gift from Yoshihiko Miyata) and subcloning into pEGFP-C1 using SalI and SacII. Kinase-dead GFP-ICK and MOK were generated using site-directed mutagenesis to change Lys 33 and 35, respectively, to Met. GFP-BBS8 was generated by amplification of the BBS8 ORF from mouse BBS8 cDNA clone (IMAGE 4527657) and subcloning into pEGFP-C1 using KpnI and ApaI. CFP-centrin-2 was generated by amplification of the centrin-2 ORF from IMCD-3 cDNA and subcloning into pECFP-N1 using KpnI and BamHI. The coding sequence of mouse KIF3B (IMAGE clone 8862410) was subcloned into the pmCit-C1 vector (equivalent to pEGFP-C1 except that EGFP was replaced by mCitrine) using XhoI and EcoRI restriction sites. All constructs were confirmed by sequencing. The mCit-KIF3B construct contained a single mutation (Val 34 to Ala) which does not change the motility of KIF3B (KJV, unpublished). KIF17-mCit has been described previously [35]. ICKsh #01 (target sequence: CACAACCACGAGGCGGTGTAA), ICKsh #02 (CCAGTGAAATTGACACAATTT), MOKsh #01 (CTGGTTCTCTTGCACTAATAT), MOKsh #02 (GCCGGAGAATATCCTAGTAAA) and control shRNA were obtained from the TRC lentivirus-based shRNA library (Sigma).

### Antibodies

Primary antibodies: mouse monoclonal anti-acetylated tubulin (Sigma; immunofluorescence (IF), 1∶1,000), rabbit polyclonal anti-ICK (Gift from Zheng Fu; Western blot (WB), 1∶500), rabbit polyclonal anti-MOK (Cosmo Bio Co., Ltd.; WB, 1∶1,000), and anti-actin (Millipore; WB, 1∶5,000). Secondary antibodies: alexa594-conjugated anti-mouse (Invitrogen; IF, 1∶1,000), HRP-conjugated anti-rabbit (Dako; WB, 1∶5,000), and HRP-conjugated anti-mouse (Amersham; WB, 1∶10,000).

### Immunofluorescence and microscopy

IMCD-3 cells were fixed with 4% PFA, permeabilized with 0.15% Triton X-100 in PBS, and blocked with blocking buffer (1% BSA and 0.05% Tween-20 in 1x PBS) for 45 minutes at room temperature (RT). Cells were incubated with primary antibodies (in blocking buffer) for 1 hour at RT, and washed three times with PBS tween (0.05% Tween-20 in 1x PBS). Cells were incubated with fluorescent-conjugated secondary antibodies (in blocking buffer) for 45 minutes at RT, and washed three times with PBS tween. After the washing steps the cells were washed with 70% ethanol for 1 minute and 100% ethanol for 1 minute. The samples were air-dried and mounted on a microscope slide with mounting solution (20 mM Tris HCl pH 8, 0.2 M DABCO, 90% glycerol). Subcellular localization studies and cilia length measurements were performed using a Zeiss Imager Z1 microscope with a 63x (NA 1.4) objective.

### IFT speed measurements

Clonal IMCD-3 cells stably expressing mCit-KIF3B, IFT43-YFP, GFP-BBS8, IFT20-GFP, KIF17-mCit, GFP-ICK or GFP-MOK were grown on 18 mm cover slips. Prior to analysis glass slides were inversed onto a 24 mm cover slip and placed in a live-cell imaging chamber. Time-lapse movies were acquired on a spinning-disc microscope (CSU-X1-A1; Yokogawa) equipped with 100×1.49 NA oil objective (Nikon) and an EMCCD camera (QuantEM 512SC; Roper Scientific), installed on an inverted research microscope (Eclipse Ti-E; Nikon), and controlled with MetaMorph 7.5 software (Molecular Devices). To determine the IFT particles' velocities, kymographs were generated in ImageJ with the Kymograph plugin, written by J. Rietdorf.

### Retroviral expression

Third-generation lentiviruses were packaged in HEK293T cells by transient co-transfection, with Lipofectamine 2000 (Invitrogen), of pMDg/RRE, pRSVREV, pMD.9, and pLKO.1-puro containing non-target control shRNA, ICKsh #01, ICKsh #02, MOKsh #01, or MOKsh #02. IMCD-3 cells growing at 40% confluency were transduced with lentiviruses. Forty-eight hours after transduction, cells were serum-starved for 48 hours in the presence of 5 µg/ml puromycin and harvested for protein extraction or used for microscopy.

### Western Blot Analysis

IMCD-3 cells were harvested in lysis buffer (50 mM Tris pH 6.8, 5 mM EDTA, 5% glycerol, 2% SDS, 1% β-mercaptoethanol, and Protease Inhibitor Cocktail (Roche)). Lysates were centrifuged at 13.200 rpm for 1 minute at 4°C. Supernatants were collected, and 1x Laemmli loading buffer was added. Protein samples were separated using SDS-PAGE and transferred to a nitrocellulose transfer membrane (Whatman). Membranes were probed with primary antibodies, washed three times with PBST (0.25% Tween-20 in 1x PBS), probed with HRP-conjugated secondary antibodies, washed three times with PBST, and exposed to chemiluminescence reagent (Amersham). Chemiluminescence was detected with Alliance 2.7 (UVItec).

### Statistical analysis

P values were derived from one-way ANOVA analysis, followed by a Bonferroni post-hoc test, using SPSS.

## Results

### ICK and MOK localize to cilia

**Figure 1:**
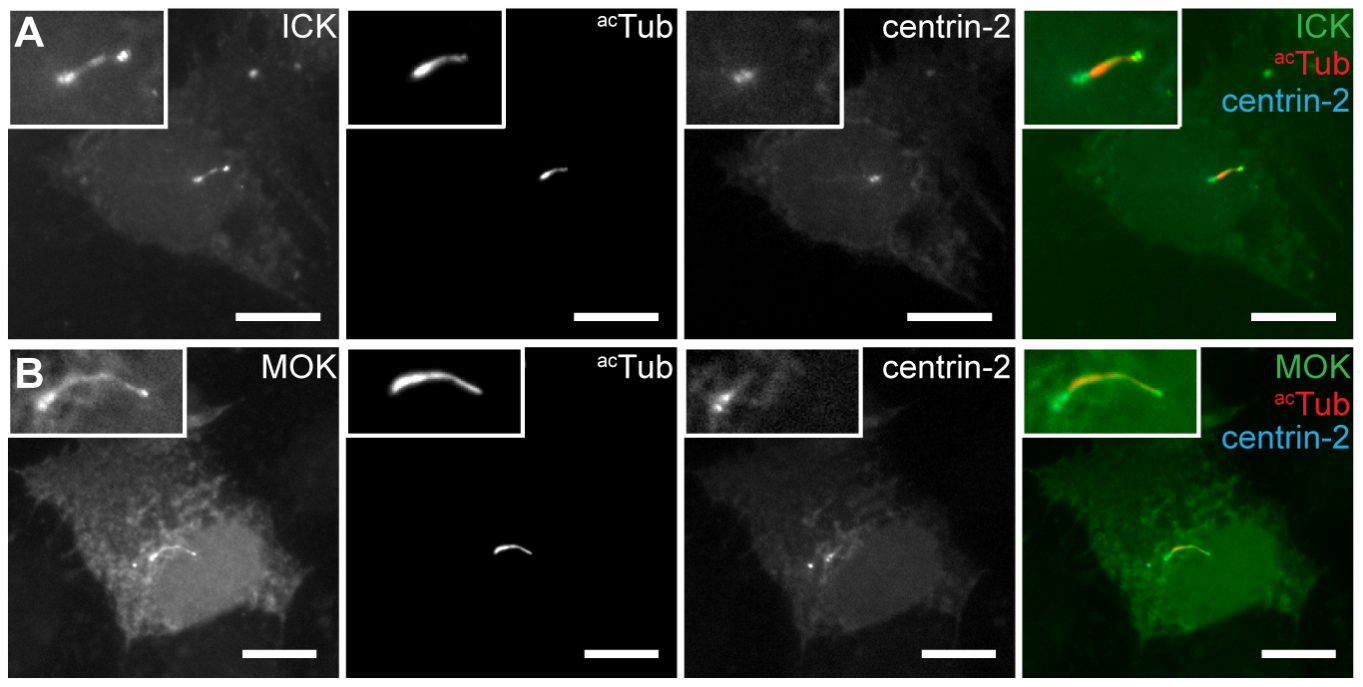
ICK and MOK localize to the primary cilium. IMCD-3 cells expressing (A) GFP-ICK and CFP-centrin-2, or (B) GFP-MOK and CFP-centrin-2, serum-starved for 48 hours and immunostained for acetylated tubulin (acTub). Insets show enlargements of the region containing the cilium. Scale bars 10 µm.

RT-PCR analysis of dividing or serum-starved IMCD-3 cells showed expression of ICK and MOK, but not of MAK in these cells (Figure S1). To investigate whether ICK and MOK localize to the cilium, we expressed N- and C-terminal GFP-fusion constructs of ICK and MOK in IMCD-3 cells. GFP-ICK, GFP-MOK and C-terminally tagged ICK and MOK localized to the primary cilium in serum-starved IMCD-3 cells (Figure 1A and B and data not shown). In addition, we observed two spots at the ciliary base, which co-localized with the centrosomal marker centrin-2 (Figure 1A and B). ICK and MOK also localized to the nucleus, in line with previous observations [36], [37].

### ICK and MOK regulate cilium length

**Figure 2:**
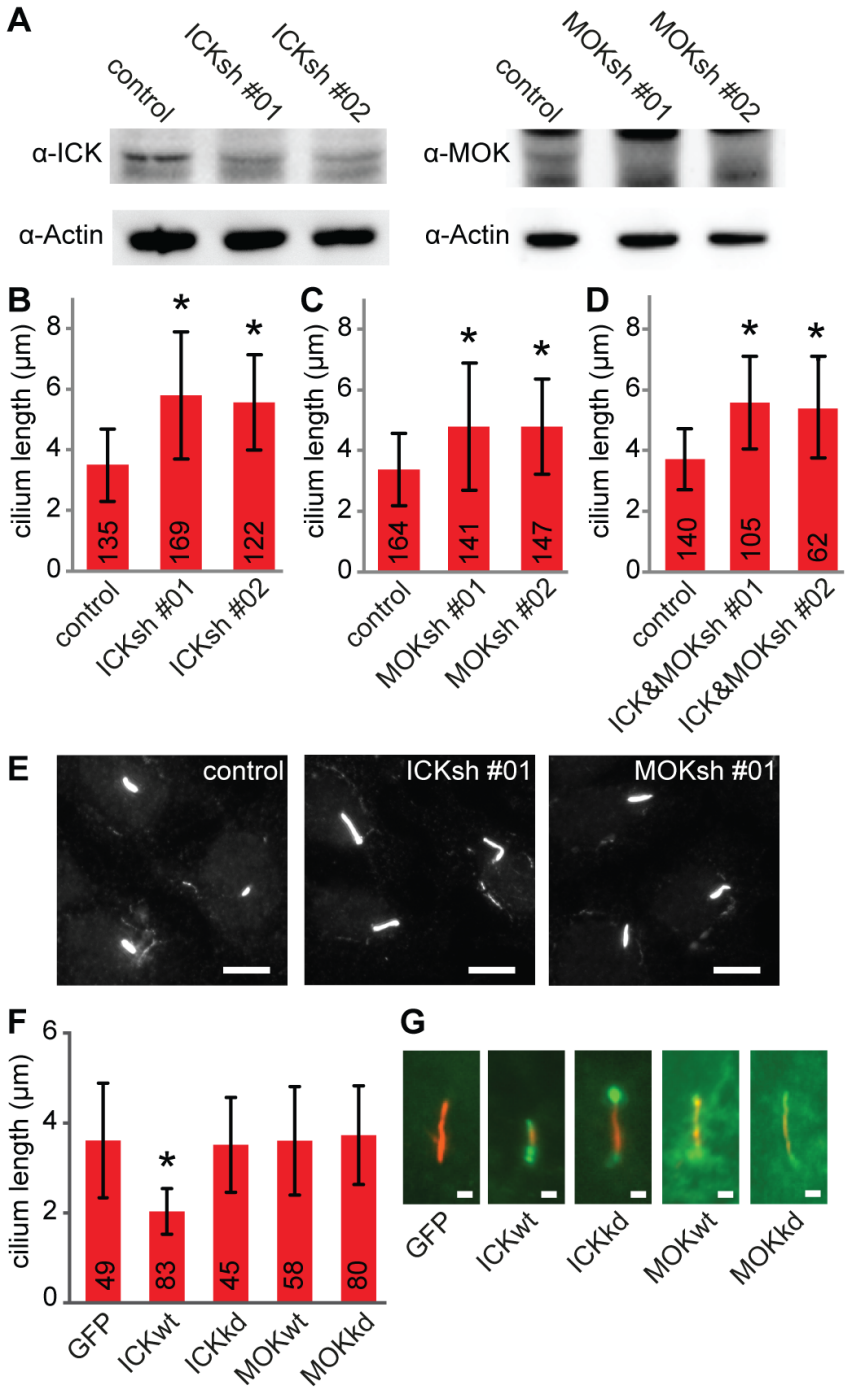
ICK and MOK control cilium length. (A) Immunoblot of cell lysates of IMCD-3 cells transduced with shRNA constructs targeting ICK and MOK. Actin was used as loading control. (B) Average length of primary cilia of IMCD-3 cells depleted of ICK. (C) Average length of primary cilia of IMCD-3 cells depleted of MOK. (D) Average length of primary cilia of IMCD-3 cells depleted of ICK and MOK, combining ICKsh #01 and MOKsh #01 or ICKsh #02 and MOKsh #02. (E) Immunofluorescence images of IMCD-3 cells expressing control shRNA, ICKsh #01, or MOKsh #01 stained with anti-acetylated tubulin. Scale bar 10 µm. (F) Average length of primary cilia of IMCD-3 cells overexpressing GFP-C1, wild type (wt) or kinase-dead (kd) GFP-ICK, or GFP-MOK. (G) Immunofluorescence images of IMCD-3 cells expressing GFP-C1, wt or kd GFP-ICK, or GFP-MOK stained with anti-acetylated tubulin. Scale bar 1 µm. Numbers in the red bars indicate number of cilia measured. Data were obtained in at least 2 independent experiments. Statistically significant differences (p<0.001) compared to control cells are indicated with a black asterisk. Error bars indicate SD.

To determine whether ICK and/or MOK regulate cilium length, we reduced ICK and MOK levels in IMCD-3 cells. Knockdown of ICK using two non-overlapping lentiviral shRNA's, ICKsh #01 and ICKsh #02, effectively reduced ICK expression (Figure 2A). Measurement of cilium length showed that cilia of IMCD-3 cells depleted of ICK were ∼60% longer than those of control cells (Figure 2B and E). Two independent lentiviral shRNA's, MOKsh #01 and MOKsh #02, that reduced MOK expression resulted in a ∼40% elongation of cilium length (Figure 2A, C and E). Knockdown of both ICK and MOK did not result in an additional increase of cilium length, but yielded cilia with a length comparable to that after knockdown of only ICK (Figure 2D). Neither knockdown of ICK nor MOK affected the percentage of IMCD-3 cells that formed cilia after 48 hours of serum starvation (Figure S2).

In addition, we analyzed the effect of overexpression of N-terminal GFP-fusions of wild type and kinase-dead (kd) ICK and MOK, on cilium length. To generate kinase-dead mutant constructs, we replaced an essential lysine in the ATP-binding pocket at position 33 in ICK and position 35 in MOK by methionine [25]. IMCD-3 cells transfected with GFP-ICK had ∼40% shorter cilia compared to cells transfected with GFP (Figure 2F and 2G), whereas overexpression of kinase-dead GFP-ICK did not affect cilium length (Figure 2F and G), indicating that kinase activity of ICK is necessary for its negative regulation of cilium length. Overexpression of wild type or kinase-dead GFP-MOK did not affect cilium length (Figure 2F and G).

Taken together, our data indicate that ICK and MOK negatively regulate cilium length in IMCD-3 cells and that this regulation by ICK requires its kinase function.

### Live imaging of five IFT proteins including KIF3B and KIF17 suggests they are transported by the same machinery

To determine whether elongation of cilium length caused by depletion of ICK and MOK is also accompanied by changes in IFT we set up live imaging of several IFT proteins. Thus far, only IFT20 and IFT88 have been shown to undergo IFT in mammalian cells [13], [34], [38]. To visualize at least one component of each IFT subcomplex, we generated clonal lines stably expressing fluorescent protein fusions of the kinesin-II subunit mCit-KIF3B, the complex A protein IFT43-YFP, the complex B protein IFT20-GFP and the BBSome subunit GFP-BBS8. In *C. elegans*, IFT is mediated by an additional kinesin, OSM-3 [39]. KIF17, the vertebrate homologue of OSM-3, localizes to cilia, but its function in IFT is not entirely understood [40], [41]. Using immunofluorescence, we confirmed that KIF17 is expressed in IMCD-3 cells and localizes to primary cilia (Figure S3). Therefore, we also generated a clonal line stably expressing KIF17-mCit.

**Figure 3:**
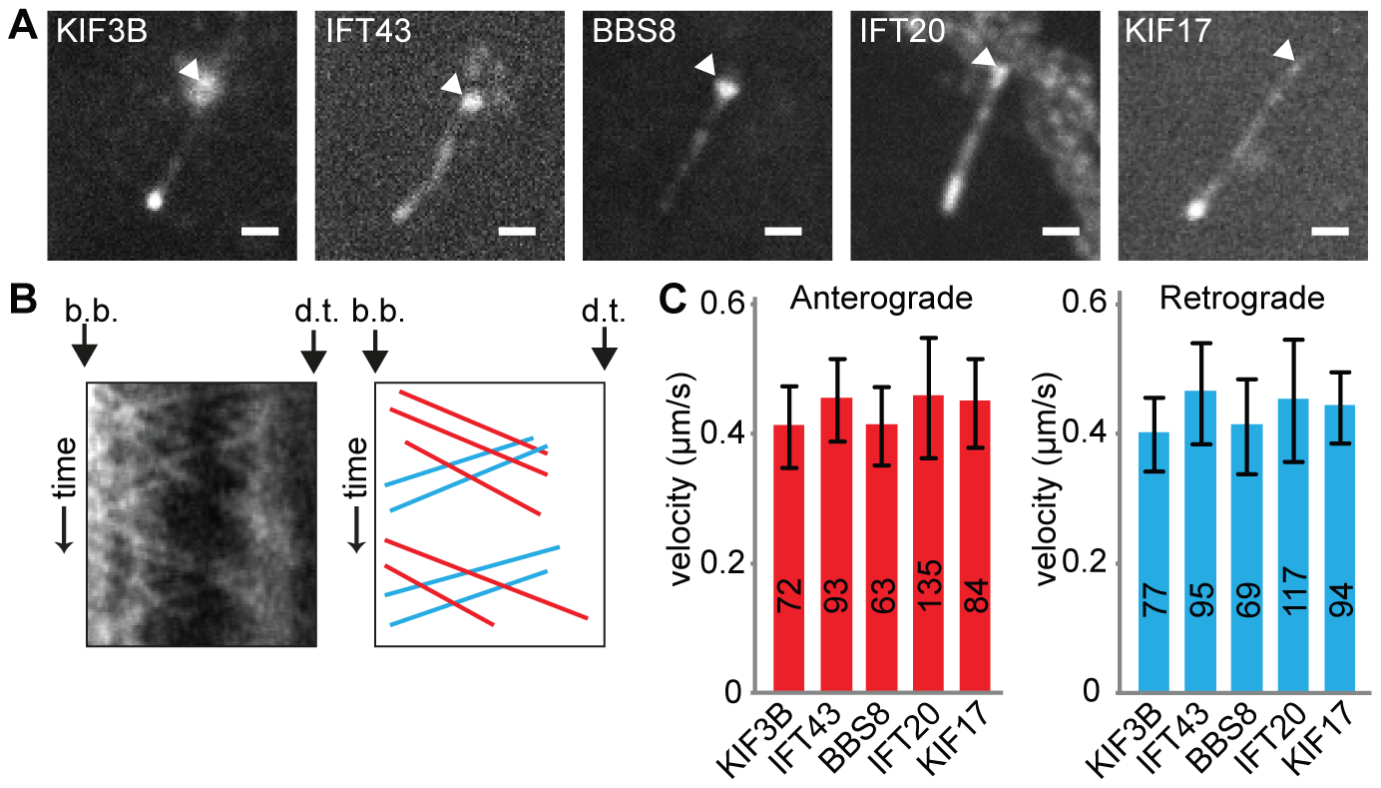
Live imaging of fluorescently tagged components of the IFT machinery. (A) Fluorescence images of the cilia of IMCD-3 cells expressing mCit-KIF3B, IFT43-YFP, GFP-BBS8, IFT20-GFP, and KIF17-mCit. The basal body is indicated with an arrowhead. Scale bar 1 µm. (B) Representative kymograph of IFT43-YFP in cilia of control cells. The basal body (b.b.) and the distal tip (d.t.) of the cilium are indicated. In the corresponding drawing, anterograde trajectories are shown in red and retrograde trajectories are shown in blue. (C) Average anterograde and retrograde velocities of IFT components in control cells. Error bars indicate SD. Numbers in the bars indicate number of particles analyzed.

All five IFT markers localized to primary cilia (Figure 3A), and moved in both anterograde and retrograde directions along the axoneme (Figure 3B and Movies S1 to S5). In IMCD-3 cells expressing a control shRNA, all five IFT markers moved at the same speeds; 0.40–0.45 µm/s in the anterograde direction and 0.39–0.46 µm/s in the retrograde direction, suggesting that all five proteins are transported by the same machinery (Figure 3B and C). These speeds are in agreement with previously reported speeds of mammalian IFT proteins which vary between 0.3–0.7 µm/s in anterograde and retrograde directions [13], [34], [38].

### ICK regulates IFT speeds

**Figure 4:**
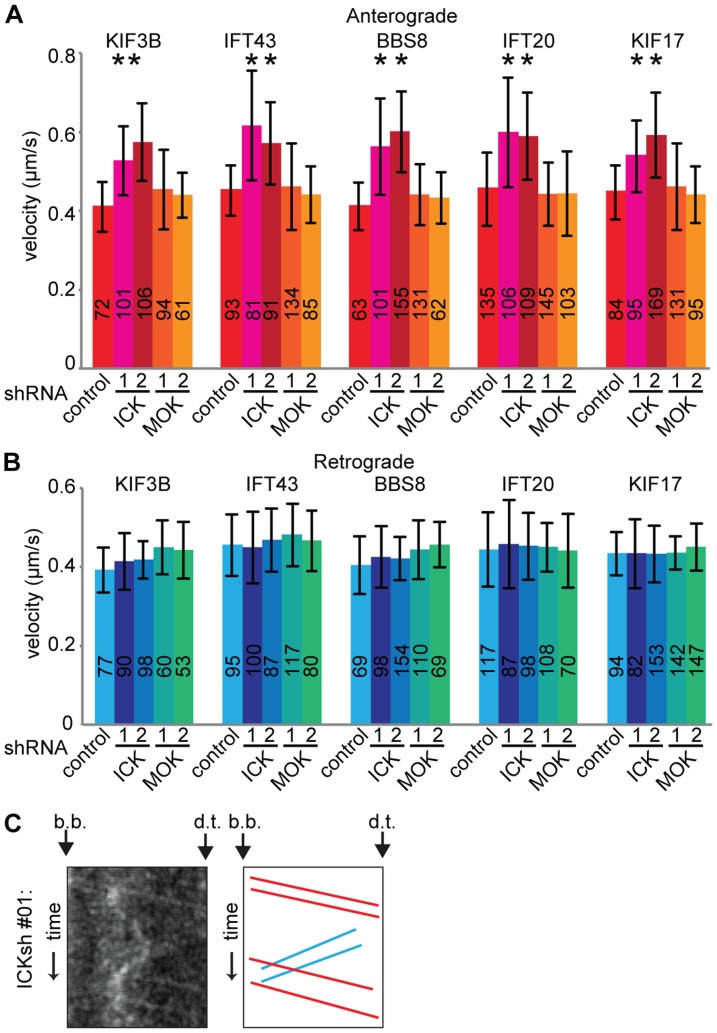
Depletion of ICK results in increased anterograde IFT velocity. (A) Average anterograde velocities of IFT components in control cells, and cells depleted of ICK or MOK using two independent shRNA constructs for each. Statistically significant differences (p<0.001) compared to the velocity of the same IFT component in control cells are indicated with a black asterisk. (B) Average retrograde velocities of IFT components in control cells, and cells depleted of ICK and MOK. (C) Representative kymograph of IFT43-YFP in cells depleted of ICK. The basal body (b.b.) and the distal tip (d.t.) of the cilium are indicated. In the corresponding, drawing anterograde trajectories are shown in red and retrograde trajectories are shown in blue. Error bars indicate SD. Numbers in the bars indicate number of particles analyzed.

To analyze whether ICK or MOK regulate IFT, we reduced the expression of ICK in the clonal lines expressing each of the five IFT markers. The average anterograde velocities of all tested IFT markers were significantly increased by approximately ∼35% in cells depleted of ICK (Figure 4A and C). In contrast, retrograde velocities were not affected (Figure 4B and C). Depletion of MOK did not affect anterograde or retrograde IFT velocities (Figure 4A and B).

**Figure 5:**
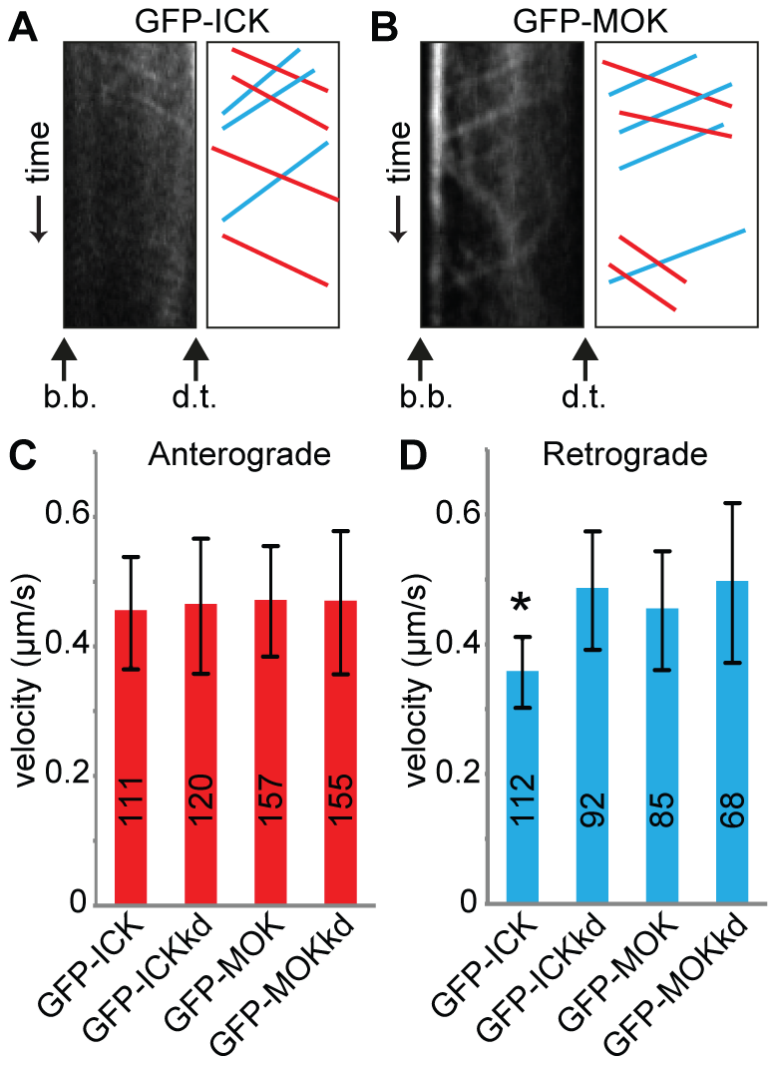
Overexpression of ICK results in decreased retrograde IFT velocity. (A) Representative example of kymograph of GFP-ICK in IMCD-3 cells. The basal body (b.b.) and the distal tip (d.t.) of the cilium are indicated. In the corresponding drawing anterograde trajectories are shown in red, and retrograde trajectories are shown in blue. (B) Representative example of kymograph of GFP-MOK in IMCD-3 cells. The basal body (b.b.) and the distal tip (d.t.) of the cilium are indicated. In the corresponding drawing anterograde trajectories are shown in red, and retrograde trajectories are shown in blue. (C) Average anterograde velocities of wt or kd GFP-ICK or GFP-MOK. (D) Average retrograde velocities of wt or kd GFP-ICK or GFP-MOK. The velocity of GFP-ICK is statistically significantly different (p<0.001) from those of GFP-ICKkd and wt or kd GFP-MOK (indicated with a black asterisk). Error bars indicate SD. Numbers in the bars indicate number of particles analyzed.

In addition, we analyzed the effect of overexpression of ICK or MOK on IFT. Since we observed GFP-tagged ICK and MOK in cilia, we surmised that these proteins are transported by the IFT machinery. We generated stable IMCD-3 cell lines expressing GFP tagged ICK, MOK or kinase dead versions of these proteins and indeed found that these GFP-tagged proteins move inside the cilium in anterograde and retrograde directions (Figure 5A and B, Movie S6 and S7). In the anterograde direction, GFP-ICK and GFP-MOK and kinase-dead versions of these proteins moved on average at 0.45–0.47 µm/s, similar to fluorescently tagged components of the IFT machinery (Figure 5C). In the retrograde direction, three of the four constructs moved at a speed similar to the IFT machinery, on average between 0.45 and 0.49 µm/s (Figure 5D). Interestingly, wild type GFP-ICK moved significantly slower in the retrograde direction, at 0.36±0.05 µm/s (mean ± sd, p<0.001 compared to GFP-ICKkd, GFP-MOK or GFP-MOKkd). These results suggest that both ICK and MOK are part of, or transported by the IFT machinery. In addition, overexpression of ICK reduces retrograde IFT speed.

### ICK and MOK interact with cAMP signaling in the regulation of cilium length and IFT

**Figure 6:**
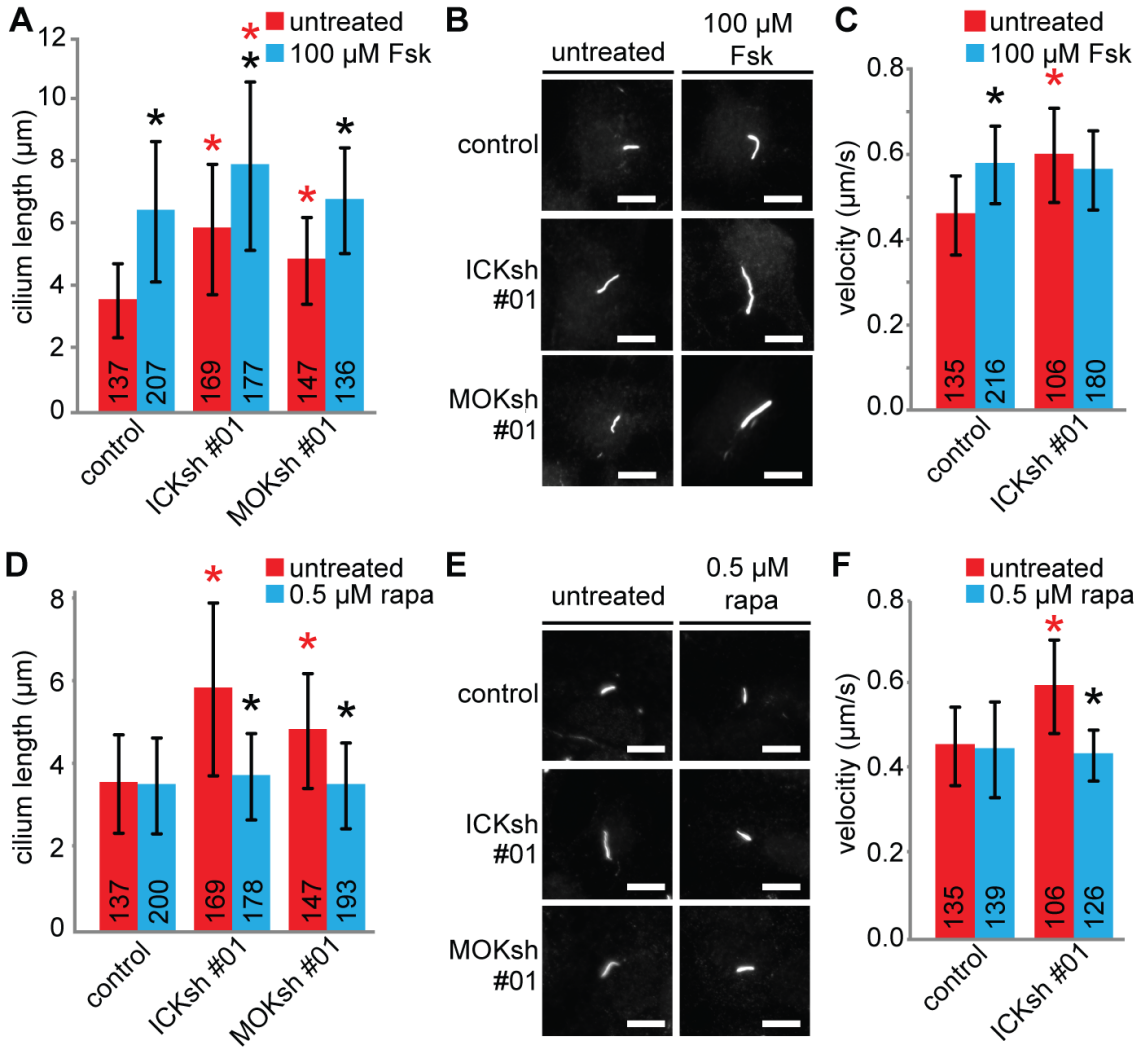
Cilium length regulation by ICK and MOK interacts with cAMP and mTORC1 signaling. (A) Average lengths of primary cilia of IMCD-3 cells expressing a control shRNA, ICKsh #01, or MOKsh #01, untreated or treated with 100 µM forskolin (Fsk) for 24 hours. (B) Immunofluorescence images of IMCD-3 cells expressing a control shRNA, ICKsh #01, or MOKsh #01, untreated or forskolin-treated, stained with anti-acetylated tubulin. (C) Average anterograde velocity of IFT20-GFP in IMCD-3 control cells, and cells depleted of ICK, untreated and forskolin-treated. (D) Average lengths of primary cilia of IMCD-3 cells expressing a control shRNA, ICKsh #01, or MOKsh #01, untreated or treated with 0.5 µM rapamycin (rapa) for 24 hours. (E) Immunofluorescence images of IMCD-3 cells expressing a control shRNA, ICKsh #01, or MOKsh #01, untreated or rapamycin-treated, stained with anti-acetylated tubulin. (F) Average anterograde velocity of IFT20-GFP in IMCD-3 control cells, and cells depleted of ICK, untreated and rapamycin-treated. Statistically significant differences (p<0.001) compared to untreated cells are indicated with a black asterisk, and compared to the control shRNA are indicated with a red asterisk. Error bars indicate SD. Numbers in the bars indicate number of cilia (A and D) or particles (C and F) analyzed. Scale bar, 10 µm.

Previous studies have shown that cilium length and IFT can be regulated by several signaling molecules, including cAMP levels and the mTOR pathway [13], [42], [43]. To analyze how cAMP signaling relates to the ciliary functions of ICK and MOK, we treated control cells and cells depleted of ICK or MOK with forskolin, resulting in increased cAMP, and measured cilium length and IFT velocities. Increased cAMP resulted in increased cilium length and anterograde velocity of IFT particles (Figure 6A and B), consistent with previous work [13]. Interestingly, forskolin treatment resulted in additive changes in cilium length when combined with ICK depletion but not MOK depletion (Figure 6A and B). These data suggest that ICK and the cAMP pathway independently regulate cilium length, whereas MOK and forskolin affect the same pathway. We thus tested if ICK depletion and forskolin treatment have additive effects on IFT speeds. Surprisingly, depletion of ICK and treatment with forskolin at the same time did not result in additive effects on the anterograde IFT velocity of IFT20-GFP (Figure 6C). This suggests that although ICK and cAMP act in the same pathway to regulate IFT, they have separable functions in regulating cilium length.

### The effects of ICK and MOK on cilium length and IFT require mTORC1 signaling

Cilium length and function are also regulated by the mTOR pathway. mTOR exists in at least two molecular complexes, mTORC1 and mTORC2, which are defined by the presence of either Raptor (mTORC1) or Rictor (mTORC2) [44]. Treatment of *C. reinhardtii* cells and zebrafish embryos with the mTORC1 inhibitor rapamycin resulted in shorter cilia [43]. Interestingly, ICK has been shown to phosphorylate Raptor at Thr-908, and thus likely modulates mTORC1 activity [45]. Since both mutation of this Thr to a non-phosphorylatable Ala or to a phosphomimic Glu impaired mTORC1 activity, it is not clear whether ICK activates or inactivates mTORC1 [45].

To investigate whether ICK and/or MOK interact with the mTORC1 pathway in the regulation of cilium length, we treated control cells, and cells expressing ICKsh #01 or MOKsh #01 with rapamycin. In contrast to the previously reported shortening of cilia [43], rapamycin treatment of control cells had no effect on cilium length (Figure 6D and E). However, rapamycin treatment completely suppressed the effect of ICK or MOK depletion on cilium length (Figure 6D and E). Rapamycin treatment also suppressed the effect of ICK depletion on the anterograde IFT velocity of IFT20-GFP (Figure 6F). Our data suggest that the effects of depletion of ICK and MOK on cilium length and of ICK on IFT require mTORC1 activity, which is in line with ICK acting as a mTORC1 inhibitor.

## Discussion

Here we show that mouse renal epithelial (IMCD-3) cells express two members of the small family of RCK kinases, ICK and MOK. In agreement with previous reports [12], [18]–[23], [36], [37], we found that ICK and MOK localize to the cilium, the ciliary base and the nucleus, and that they negatively regulate cilium length. The strongest effects on cilium length were obtained by ICK knock down, which increased cilium length, or overexpression, which decreased cilium length.

Our work extends the previous findings by examining the mechanisms of RCK function in ciliary length control. We show that both GFP-tagged ICK and MOK move inside cilia at speeds comparable to those of the IFT machinery, suggesting they are transported by, or part of the IFT machinery. Transport of RCKs by the IFT complex would enable tight control of IFT during ciliary length control. Indeed, we found that ICK regulates IFT speed: ICK knock down increased anterograde speed, whereas ICK overexpression reduced retrograde IFT speed. These findings are consistent with the balance point model as an increase in IFT speed should result in increased delivery of IFT particles to the cilium tip. Unfortunately, it proved to be impossible to accurately and reproducibly measure the number of IFT particles arriving at the tip or their size in our current imaging setup. Our results also suggest that retrograde transport can play a role in regulation of cilium length, consistent with effects of inactivation of the dynein heavy chain in *Chlamydomonas* [15].

Although a change in IFT can be associated with a change in cilium length, several lines of evidence indicate that regulation of cilium length does not necessarily involve changes in IFT speed and can be achieved by other mechanisms. First, knockdown of MOK affects cilium length, but does not affect IFT speeds. Second, knockdown of ICK and addition of forskolin work together to regulate IFT but each has its own additional functions in regulating cilium length.

We show that the regulation of cilium length by ICK requires its kinase function, as overexpression of a kinase dead mutant version of ICK did not affect cilium length. The kinase function of ICK likely impacts mTORC1 as the effect of knockdown of ICK on cilium length or IFT required mTORC1 signaling. This is consistent with an earlier report showing that ICK modulates mTORC1 activity [45]. Since the mTORC1 complex directly regulates protein synthesis in mammals and the level of soluble tubulin positively regulates cilia length, ICK and MOK could act as negative regulators of cilium length by suppressing the synthesis of ciliary tubulin via inhibition of mTORC1 [10], [46]. Another possibility of how ICK might control IFT is by direct phosphorylation of the kinesin-II subunit KIF3A, which seems to affect cilium formation and function in cultured mammalian cells and in zebrafish [22].

Our live imaging of IFT components provides the first direct demonstration that the kinesin-2 motors move with speeds compatible with IFT in mammalian cells. Indeed, we found that all five components of the IFT machinery tested, the kinesin-II subunit KIF3B, homodimeric kinesin KIF17, complex A protein IFT43, complex B protein IFT20 and BBSome protein BBS8, move at the same speeds in anterograde and retrograde directions, and that the anterograde speeds of all proteins are affected to a similar extent upon knockdown of ICK. These findings strongly suggest that in IMCD-3 cells, all five components of the IFT machinery tested are transported by the same machinery, including the two kinesins, kinesin-II and KIF17. The exact functions of the two kinesins in the cilia of IMCD-3 cells, for example whether they coordinate their motility as shown in *C. elegans* [39], remain to be determined. Further analysis of the mammalian IFT machinery is required to decipher the composition of the IFT complexes.

In conclusion, both ICK and MOK modulate cilium length and add to the complexity required to achieve the variety in lengths and morphologies of cilia that are probably necessary for different cell-specific functions. Inappropriate elongation of cilia hinders the biological processes in which cilia function [43], [47]. Not surprisingly, several ciliopathies are associated with defects in cilia length control. For example, MAK, negative regulator of cilia length and close relative of ICK and MOK, has been linked to the retina-specific ciliopathy retinitis pigmentosa [30], [31]. In addition, recently generated *Ick* knock-out mice show developmental defects that resemble those observed in the human syndrome endocrine-cerebro-osteodysplasia [22], [23]. Also Juvenile Cystic Kidney Disease and Meckel–Gruber Syndrome have been linked to the long cilia phenotype [48], [49]. Further research is necessary to gain more insight in the signal transduction pathways that regulate cilium length as well as the role of IFT in achieving these differences in cilium length.

## Supporting Information

Figure S1 **ICK and MOK are expressed in IMCD-3 cells.** MAK, ICK, and MOK expression in dividing (S) or serum-starved (G0) IMCD-3 cells visualized by RT-PCR.
(TIF)

Figure S2 **Knock down of ICK or MOK does not affect cilia formation.** Percentage of ciliated cells in IMCD-3 cells depleted of ICK or MOK. Numbers indicate numbers of cells analyzed, error bars represent SD. These results are based on 15 pictures of transfected cells, from one experiment.
(TIF)

Figure S3 **Endogenous KIF17 localizes to cilia of IMCD-3 cells.** IMCD-3 cells, serum-starved for 48 hours, were immunostained for KIF17 and acetylated tubulin. Insets show enlargements of the region containing the cilium. Scale bars 10 µm.
(TIF)

Movie S1 **mCit-KIF3B motility in a cilium.** Time-lapse video of an IMCD-3 cell expressing mCit-KIF3B. The video displays 20 frames per second for ∼6 seconds (original video ∼36 seconds). Scale bar 1 µm.
(AVI)

Movie S2 **IFT43-YFP motility in a cilium.** Time-lapse video of an IMCD-3 cell expressing IFT43-YFP. The video displays 20 frames per second for ∼6 seconds (original video ∼40 seconds). Scale bar 1 µm.
(AVI)

Movie S3 **GFP-BBS8 motility in a cilium.** Time-lapse video of an IMCD-3 cell expressing GFP-BBS8. The video displays 20 frames per second for ∼6 seconds (original video ∼49 seconds). Scale bar 1 µm.
(AVI)

Movie S4 **IFT20-GFP motility in a cilium.** Time-lapse video of an IMCD-3 cell expressing IFT20-GFP. The video displays 20 frames per second for ∼6 seconds (original video ∼37 seconds). Scale bar 1 µm.
(AVI)

Movie S5 **KIF17-mCit motility in a cilium.** Time-lapse video of an IMCD-3 cell expressing KIF17-mCit. The video displays 20 frames per second for ∼6 seconds (original video ∼39 seconds). Scale bar 1 µm.
(AVI)

Movie S6 **GFP-ICK motility in a cilium.** Time-lapse video of an IMCD-3 cell expressing GFP-ICK. The video displays 20 frames per second for ∼6 seconds (original video ∼41 seconds). Scale bar 1 µm.
(AVI)

Movie S7 **GFP-MOK motility in a cilium.** Time-lapse video of an IMCD-3 cell expressing GFP-MOK. The video displays 20 frames per second for ∼6 seconds (original video ∼38 seconds). Scale bar 1 µm.
(AVI)
