# Supplementary figures and images for: Regulation of Cilium Length and Intraflagellar Transport by the RCK-Kinases ICK and MOK in Renal Epithelial Cells
Source: PLoS One. 2014 Sep 22;9(9):e108470. doi: 10.1371/journal.pone.0108470 (PMC4171540; doi:10.1371/journal.pone.0108470)

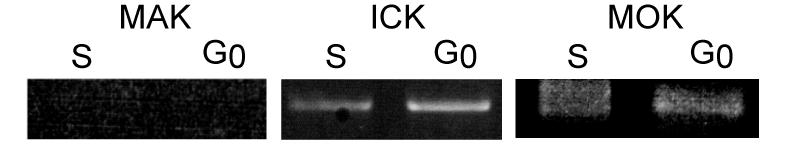

Supplement: Figure S1 — ICK and MOK are expressed in IMCD-3 cells. MAK, ICK, and MOK expression in dividing (S) or serum-starved (G0) IMCD-3 cells visualized by RT-PCR. (TIF) [file pone.0108470.s001.tif]

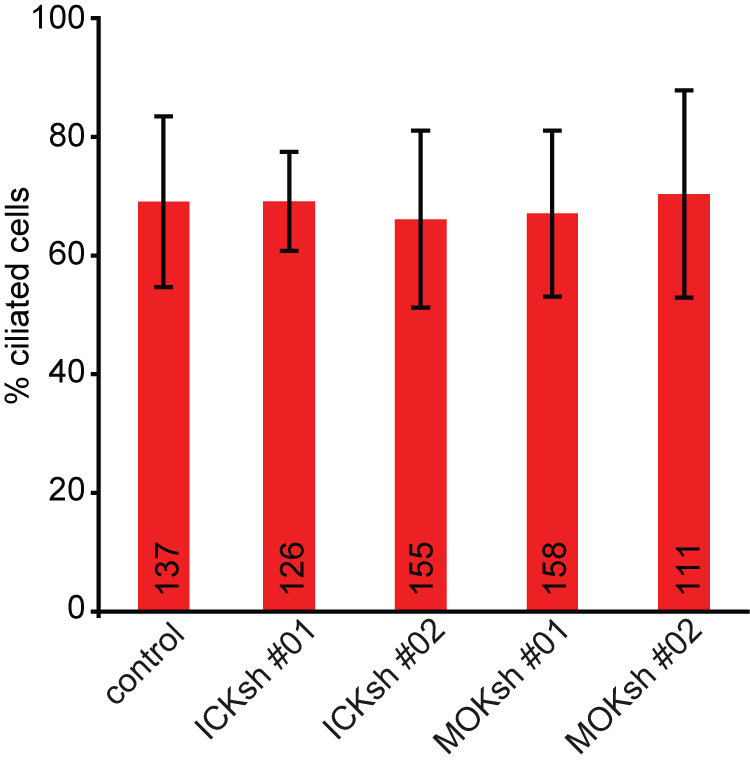

Supplement: Figure S2 — Knock down of ICK or MOK does not affect cilia formation. Percentage of ciliated cells in IMCD-3 cells depleted of ICK or MOK. Numbers indicate numbers of cells analyzed, error bars represent SD. These results are based on 15 pictures of transfected cells, from one experiment. (TIF) [file pone.0108470.s002.tif]

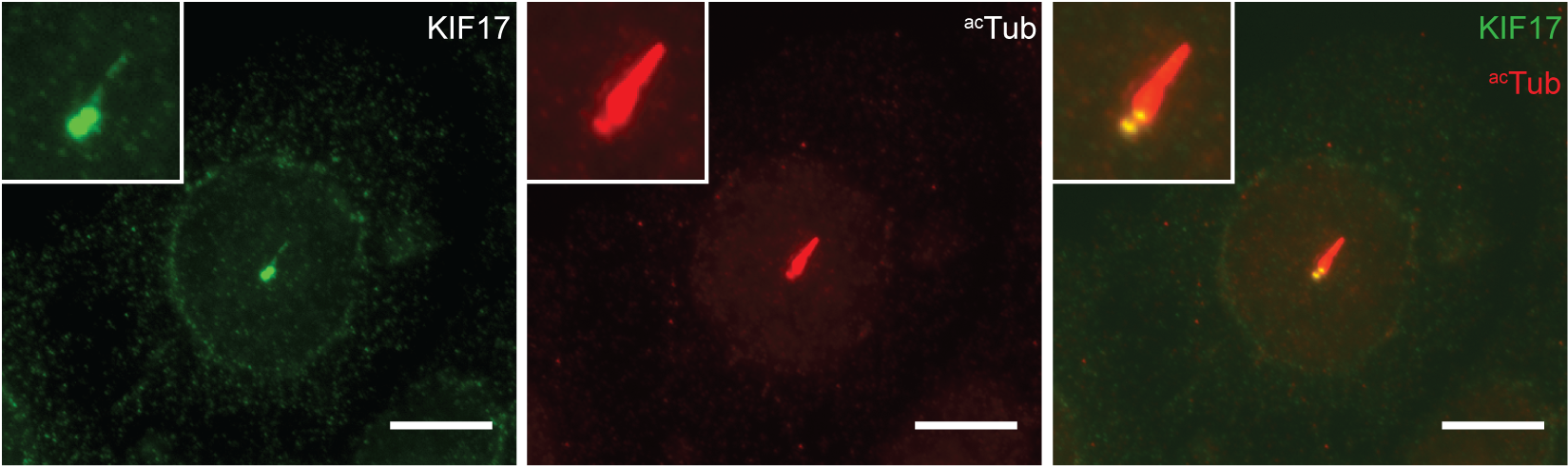

Supplement: Figure S3 — Endogenous KIF17 localizes to cilia of IMCD-3 cells. IMCD-3 cells, serum-starved for 48 hours, were immunostained for KIF17 and acetylated tubulin. Insets show enlargements of the region containing the cilium. Scale bars 10 µm. (TIF) [file pone.0108470.s003.tif]
